# Vaccine Effectiveness against Medically Attended Laboratory-Confirmed Influenza in Japan, 2011–2012 Season

**DOI:** 10.1371/journal.pone.0088813

**Published:** 2014-02-13

**Authors:** Motoi Suzuki, Le Nhat Minh, Hiroyuki Yoshimine, Kenichiro Inoue, Lay Myint Yoshida, Konosuke Morimoto, Koya Ariyoshi

**Affiliations:** 1 Department of Clinical Medicine, Institute of Tropical Medicine, Nagasaki University, Nagasaki, Japan; 2 Inoue Hospital, Shunkaikai, Nagasaki, Japan; 3 Department of Paediatric Infectious Diseases, Institute of Tropical Medicine, Nagasaki University, Nagasaki, Japan; University Hospital San Giovanni Battista di Torino, Italy

## Abstract

The objective of this study was to estimate influenza vaccine effectiveness (VE) against medically attended, laboratory-confirmed influenza during the 2011–2012 season in Japan using a test-negative case-control study design. The effect of co-circulating non-influenza respiratory viruses (NIRVs) on VE estimates was also explored. Nasopharyngeal swab samples were collected from outpatients with influenza-like illnesses (ILIs) in a community hospital in Nagasaki, Japan. Thirteen respiratory viruses (RVs), including influenza A and B, were identified from the samples using a multiplex polymerase chain reaction. The difference in VE point estimates was assessed using three different controls: ILI patients that tested negative for influenza, those that tested negative for all RVs, and those that tested positive for NIRVs. The adjusted VE against medically attended, laboratory-confirmed influenza using all influenza-negative controls was 5.3% (95% confidence interval [CI], −60.5 to 44.1). The adjusted VEs using RV-negative and NIRV-positive controls were −1.5% (95% CI, −74.7 to 41) and 50% (95% CI, −43.2 to 82.5), respectively. Influenza VE was limited in Japan during the 2011–2012 season. Although the evidence is not conclusive, co-circulating NIRVs may affect influenza VE estimates in test-negative case-control studies.

## Introduction

The effectiveness of the seasonal influenza vaccine differs between influenza seasons [1]. The vaccine strains do not always match the circulating strains because of antigenic drift [2]. Additionally, influenza vaccine effectiveness (VE) varies between countries because of the difference in the circulating strains and population characteristics [1]. Therefore, for effective control of influenza, country-specific influenza VE must be monitored every season [3], [4].

Recently, the test-negative case-control study design has been widely used to estimate influenza VE [3]. In this study design, samples are collected from patients with influenza-like illnesses (ILIs), and the influenza VE is estimated by comparing the vaccination status of patients who test positive for influenza with that of those who test negative, including non-influenza respiratory virus (NIRV) cases [5]. Although this study design allows for reliable influenza VE estimates, non-specific immunity induced by influenza infection may have an effect on the estimates [6]. It has been hypothesised that influenza infection induces a short-term, non-specific immunity and reduces the risk of subsequent NIRV infections. Therefore, people who are vaccinated for influenza and are less likely to be naturally infected with influenza may be at a higher risk of NIRV infections [6], [7]. If this association is true, NIRV-positive cases are more likely to have been vaccinated for influenza; thus, influenza VE estimates in test-negative case-control studies using the influenza-negative controls, including NIRV-positive cases, may overestimate the true VE, but supporting evidence is limited.

Recent test-negative case-control studies in European countries have demonstrated that influenza VE against laboratory-confirmed influenza A in the 2011–2012 season was 29% (95% confidence interval [CI], −26 to 60) in Spain [8], 23% (95% CI, −10 to 47) in the UK [4], and 25% (95% CI, −6 to 47) among vaccination target groups in eight EU member states [9]. However, none of these studies considered the effect of NIRVs on influenza VE estimates, and none of the influenza VE estimates in the 2011–2012 season have been reported from Asian countries, including Japan.

In our previous study conducted in the 2010–11 season, we demonstrated that the test-negative case-control study using RIDT results provided rapid estimates of influenza VE [10]. In the current study, we estimated influenza VE of the trivalent inactivated vaccine (TIV) against medically attended, laboratory-confirmed influenza in Japan during the 2011–2012 season using multiplex PCR. We explored the difference in influenza VE point estimates using three different controls: influenza-negative controls, NIRV-positive controls, and respiratory virus (RV)-negative controls.

## Materials and Methods

### Ethics

The study was approved by the Institutional Review Board (IRB) at Inoue Hospital, Nagasaki, and the IRB of the Institute of Tropical Medicine at Nagasaki University. Our hospital doctors informed the study objectives and methods to eligible patients and their guardians verbally during their consultations. We also provided the necessary information to patients and their guardians using a standardized questionnaire sheet and a poster presentation at the outpatient department. The requirement for obtaining written consent was waived by both IRBs. Anonymized data were used for the analysis.

### Study Setting and Enrolment Criteria

Nagasaki City is located in the western part of Japan. A prospective case-control study was conducted at a middle-sized community hospital in the city, which was the study site used in our previous influenza VE study during the 2010–2011 season [10].

All patients who visited the outpatient department, presented with ILI, and had been administered the rapid influenza diagnostic test (RIDT) were eligible for the study. We modified the original case definition for ILI used in EU countries adapting to local context [11]; a case was defined as ILI if the patient showed a sudden onset of fever and at least one of the following symptoms: cough, runny nose, sore throat, headache, myalgia, or fatigue. We aimed to recruit all age groups, but the number of child cases was limited because the hospital did not have a paediatric department.

A case was excluded if 1) it didn’t meet the ILI case definition, 2) testing was performed more than five days after disease onset, 3) testing was performed within seven days after previous testing, 4) the clinical sample was lost, or 5) it presented before the week of the first PCR-confirmed influenza case.

The study period was from December 1_,_ 2011, through April 30_,_ 2012. A standardised questionnaire was distributed to all outpatients during the study period to collect epidemiological information, including clinical symptoms, the date of onset, and influenza vaccination status for the 2011–2012 season. Patients were considered “vaccinated” only if they had been vaccinated more than 14 days prior to the hospital visit; otherwise, they were considered “unvaccinated”. Patients and their caregivers were asked to fill out the questionnaire before the consultation. Demographic and clinical information was collected from electronic medical records.

In our setting, a commercial RIDT kit (RapidTesta Flu II, Sekisui Medical, Japan) was offered to every ILI patient to diagnose influenza A- and B-positive cases as a routine practice. The RIDT was performed by skilled nurses or laboratory technicians. Residual nasopharyngeal swabs were temporarily stored at −20°C in the laboratory department of the hospital after being used for the RIDT. Within a week, the samples were transported to the Institute of Tropical Medicine at Nagasaki University for storage in a deep freezer (−80°C) until they were used for further molecular tests.

### Vaccines

In Japan, all children under 13 years of age are recommended to receive 2 doses of the seasonal influenza vaccine, and others are recommended to receive one dose by the Ministry of Health, Labor and Welfare. The cost of vaccination for the elderly is partially or fully subsidized by the local government [12]. The TIVs produced by domestic manufacturers are used for influenza vaccination; no live attenuated influenza vaccine has been approved in Japan. We therefore assumed that people who reported vaccinated for influenza in the 2011–2012 season had been vaccinated with TIVs.

The World Health Organization (WHO) recommended that the vaccines for use in the 2011–2012 northern hemisphere influenza season contain A/California/7/2009(H1N1)-like, A/Perth/16/2009(H3N2)-like, and B/Brisbane/60/2008-like strains [13]. For the A/Perth/16/2009(H3N2)-like component, the A/Victoria/210/2009(H3N2) strain was used in Japan [14].

### Virus Characterisation

Viral nucleic acid was extracted using a QIA viral RNA minikit (QIAGEN Inc., Valencia, CA). The following four multiplex PCR assays were performed for each sample to detect 13 RVs: (1) influenza A/B, respiratory syncytial virus (RSV), and human metapneumovirus (HMPV); (2) human parainfluenza virus (HPIV) types 1–4; (3) human rhinovirus (HRV) and human coronavirus OC43/229E (HCoV); and (4) human adenovirus (HAdV) and human bocavirus (HBoV). Details of the multiplex PCR assays are published elsewhere [15], [16]. Briefly, reverse transcription-PCR (RT-PCR) assays were performed using the one-step RT-PCR kit from QIAGEN. Confirmatory PCR was performed using a hemi-nested PCR assay on samples that were positive in initial PCR tests; samples positive in both multiplex and hemi-nested PCRs were defined as positive. In the current study, PCR results but not RIDT results were used to define laboratory-confirmed influenza cases. HA subtyping was performed for influenza A-positive samples via RT-PCR and sequencing of the influenza HA gene using previously published methods [17].

The phylogenetic tree was constructed using the Neighbor-Joining method. HA1 sequences from reference strains used in the phylogenetic analysis were obtained from the EpiFlu database of the Global Initiative on Sharing Avian Influenza Data (GISAID).

### Data Analysis

Patients were categorised into three groups: ILI episodes that were positive for influenza A and B (influenza positive cases), negative for influenza and positive for non-influenza respiratory viruses (NIRV-positive controls), and negative for all respiratory viruses (RV-negative controls). The characteristics of the study patients were compared by outcome categories using chi-square tests, Fisher’s exact tests, and Kruskal-Wallis tests depending on the nature of the variable. The patients’ ages were categorised into four groups: 10–19 years, 20–49 years, 50–64 years, and 65 years and above. The early and late phases of the influenza season were defined as up to week 8 of 2012 and from week 9 to week 17 of 2012.

We used the test-negative case-control study design to estimate influenza VE; specifically, the cases were all ILI episodes that were positive for influenza A and/or B, and the controls were all ILI episodes that were negative for both influenza A and B (all influenza-negative controls). We also estimated the VE against influenza A by excluding the influenza B-positive cases. Influenza VE estimates were calculated as 1– the odds ratio (OR). Logistic regression models were used to estimate the unadjusted and adjusted ORs. Age group, underlying conditions, duration from onset to testing, and month of visit were included in the final models. We did not exclude patients with unknown vaccination history (<10%), but we instead coded those missing values as “unknown status” and included all patients in our analysis. To explore the difference in influenza VE estimates by control group, we conducted analyses using NIRV-positive controls and RV-negative controls.

The sensitivity and specificity of RIDT for detecting influenza were assessed considering PCR results as the gold standard. We also estimated the influenza VE using RIDT results and compared with those using PCR results. All statistical analyses were performed using STATA 11.2 (STATA Corp., USA).

## Results

**Figure 1:**
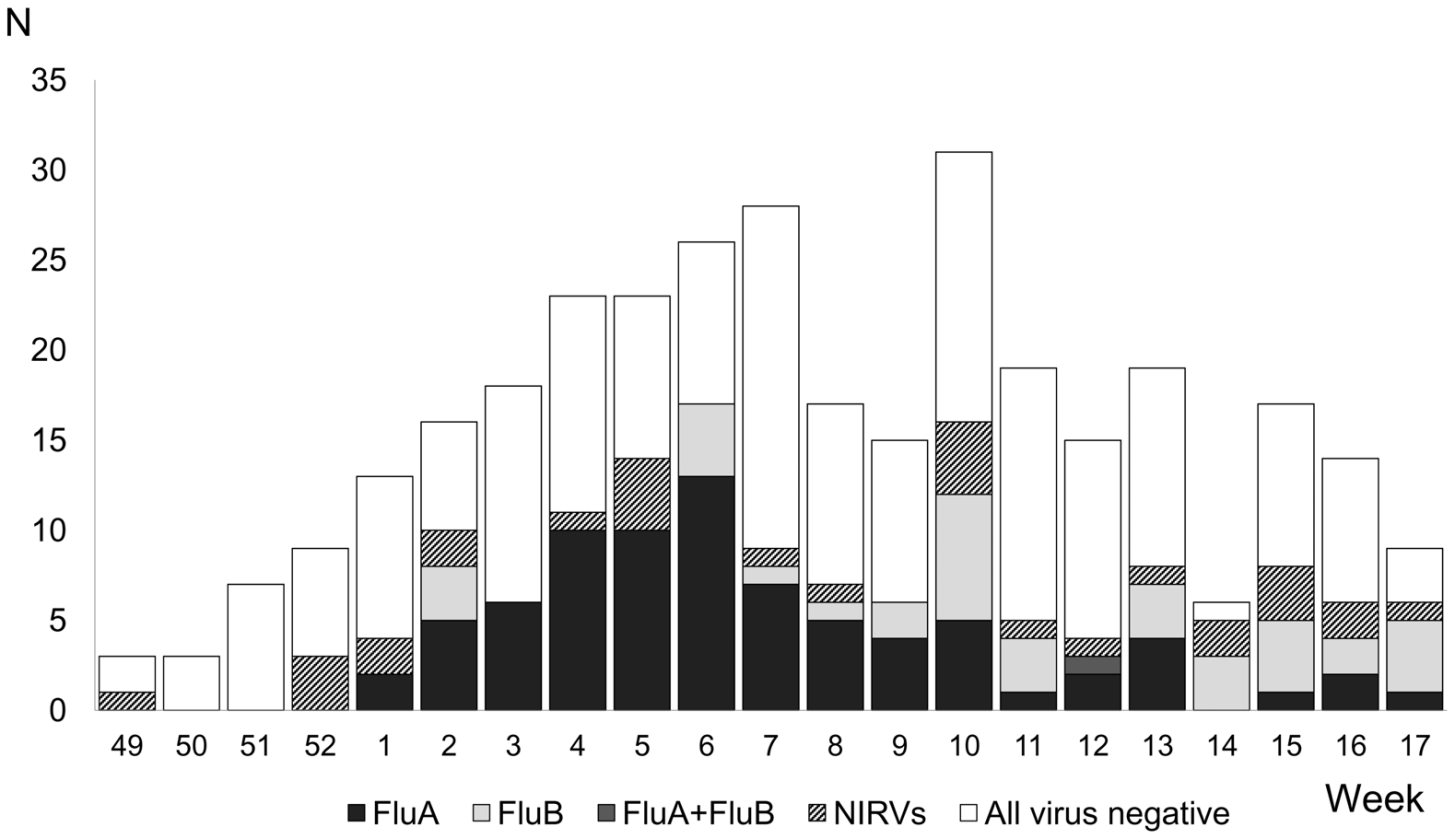
Laboratory detection of influenza and non-influenza respiratory viruses (NIRVs) by week in Nagasaki, Japan (2011–2012 season). FluA, influenza virus A; FluB, influenza virus B; NIRVs, non-influenza respiratory viruses.

Between December 2011 and April 2012, 444 ILI episodes were enrolled in the study. After the exclusion of 77 episodes that did not meet the ILI case definition, 9 episodes that were tested within 7 days after the previous testing, 9 episodes that were tested more than 5 days after symptom onset, 18 episodes associated with lost clinical samples, and 22 episodes that presented before the week of the first influenza case, 309 episodes were eligible for analyses. Among them, 78 (25.2%) were single-positive for influenza A, 37 (12%) were single-positive for influenza B, and one (0.3%) was positive for both influenza A and B (Table 1). The influenza season started in the first week of 2012 and reached its peak at the 6^th^ week (Figure 1). Influenza B was the dominant strain in the late phase (week 9 to week 17).

**Table 1:** Number of virus-positive patients among the study population (N = 309).

Viruses^a^	No. of PCR positives	
FluA	79	25.6%
FluB	38	12.3%
HRV	11	3.6%
RSV	8	2.6%
HCoV	4	1.3%
HPIV1	2	0.7%
HPIV2	2	0.7%
HMPV	1	0.3%
Dual-positive cases		
FluA+FluB	1	0.3%
FluB+HRV	1	0.3%
HRV+RSV	1	0.3%

a FluA, influenza virus A; FluB, influenza virus B; HRV, human rhinovirus; RSV, respiratory syncytial virus; HCoV, human coronavirus; HPIV, human parainfluenza virus; HMPV, human metapneumovirus.

Among the 46 influenza A-positive samples that were subtyped (58.2% of all influenza A-positive samples), all were positive for the H3N2 strain. All unsubtyped influenza A samples were negative for the A(H1N1)pdm09 strain. From the collected samples, 27 (8.7%) were positive for NIRVs; HRV was the leading virus identified, followed by RSV. One case was positive for both influenza B and HRV, and the case was classified as an influenza B-positive case. NIRVs were identified throughout the study period.

The characteristics of patients by case category are summarised in Table 2. Influenza-positive cases were younger than influenza-negative groups, and NIRV-positive cases were more frequently vaccinated for influenza than other groups, whereas other characteristics were similar across case categories.

**Table 2:** Characteristics of study participants in Nagasaki, Japan, by outcome category.

	Influenza cases	Non-influenza respiratory virus-positive controls	All respiratory virus-negative controls	
	N = 116	N = 26	N = 167	*P* value
	N (%)/Median (IQR^a^)	N (%)/Median (IQR)	N (%)/Median (IQR)	
Sex				
Female	51 (44)	15 (57.7)	73 (43.7)	0.396^b^
Male	65 (56)	11 (42.3)	94 (56.3)	
Age category				
10–19 years	25 (21.6)	3 (11.5)	21 (12.6)	0.046^c^
20–49 years	61 (52.6)	12 (46.2)	88 (52.7)	
50–64 years	17 (14.7)	3 (11.5)	18 (10.8)	
≥65 years	13 (11.2)	8 (30.8)	40 (24)	
Age (years)	31 (28.5)	35.5 (52)	37 (36)	0.013^d^
Chronic conditions				
Present	30 (25.9)	10 (38.5)	59 (35.3)	0.187^b^
Absent	86 (74.1)	16 (61.5)	108 (64.7)	
Date of OPD visit				
January 2012	31 (26.7)	8 (30.8)	43 (25.8)	0.085^c^
February 2012	40 (34.5)	3 (11.5)	47 (28.1)	
March 2012	28 (24.1)	7 (26.9)	56 (33.5)	
April 2012	17 (14.7)	8 (30.8)	21 (12.6)	
Duration of symptoms (days between onset and swabbing)				
0–1	83 (71.6)	17 (65.4)	116 (69.5)	0.807^c^
2–3	28 (24.1)	9 (34.6)	44 (26.4)	
4–5	5 (4.3)	0 (0)	7 (4.2)	
Vaccination status for the 2011/12 season				
Vaccinated	38 (32.8)	12 (46.2)	54 (32.3)	0.029^c^
Unvaccinated	74 (63.8)	10 (38.5)	95 (56.9)	
Unknown	4 (3.5)	4 (15.4)	18 (10.8)	

a Interquartile range.

b Chi-square test.

c Fisher’s exact test.

d Kruskal-Wallis test.

The unadjusted estimate of influenza VE against medically attended, laboratory-confirmed influenza was 18.3% (95% confidence interval: −34.4 to 50.3) based on the use of all influenza-negative controls (Table 3). After controlling for potential confounders, including age group, underlying condition, duration from disease onset to testing, and month of visit, the adjusted VE estimate was 5.3% (95% CI: −60.5 to 44.1). When we restricted the analysis to influenza A only, the unadjusted and adjusted VE estimates were 26.7% (95% CI: −28.1 to 58.1) and 16% (95% CI: −54.5 to 54.3), respectively. When we stratified the study season into 2 periods, the adjusted estimate of VE against influenza A was 21.7% (95% CI: −67.5 to 63.4) in the early phase (week 1 to week 8) and 8.9% (95% CI: −155.7 to 67.5) in the late phase (week 9 to week 17) (data not shown in the table).

**Table 3:** The effectiveness of the trivalent inactivated vaccine against medically attended influenza in the 2011–2012 season in Nagasaki, Japan.

	Cases (N)/controls (N)	Crude VE^a^ (95% CI^b^)	Adjusted VE^c^ (95% CI)
VE against medically attended influenza			
All Influenza-negative controls	116/193	18.3 (−34.4 to 50.3)	5.3 (−60.5 to 44.1)
All respiratory virus-negative controls	116/167	9.7 (−51.1 to 46)	−1.5 (−74.7 to 41)
Non-influenza respiratory virus-positive controls	116/26	57.2 (−8 to 83)	50 (−43.2 to 82.5)
VE against medically attended influenza A			
All Influenza-negative controls	79/193	26.7 (−28.1 to 58.1)	16 (−54.5 to 54.3)
All respiratory virus-negative controls	79/167	18.8 (−46.2 to 54.9)	8.7 (−71.6 to 51.4)
Non-influenza respiratory virus-positive controls	79/26	61.5 (−1.3 to 85.4)	53.6 (−52.6 to 85.9)

a Vaccine effectiveness.

b Confidence interval.

c All models were adjusted for age group, underlying condition, duration from illness onset to testing, and month of visit.

When controls were limited to RV-negative controls, the adjusted estimate of VE against influenza became −1.5% (95% CI: −74.7 to 41). When controls were limited to NIRV-positive controls, the adjusted VE estimate was 50% (95% CI: −43.2 to 82.5). Similar patterns were observed in the estimates of VE against influenza A.

The sensitivity and specificity of RIDT for detecting influenza A and/or B were 78.6% (95% CI: 73.9 to 83) and 95.3% (95% CI: 93 to 97.7), respectively. The unadjusted and adjusted estimates of VE against influenza using RIDT results were 14.4% (95% CI: −43.1 to 48.8) and 5.2% (95% CI: −63 to 44.9), respectively.

**Figure 2:**
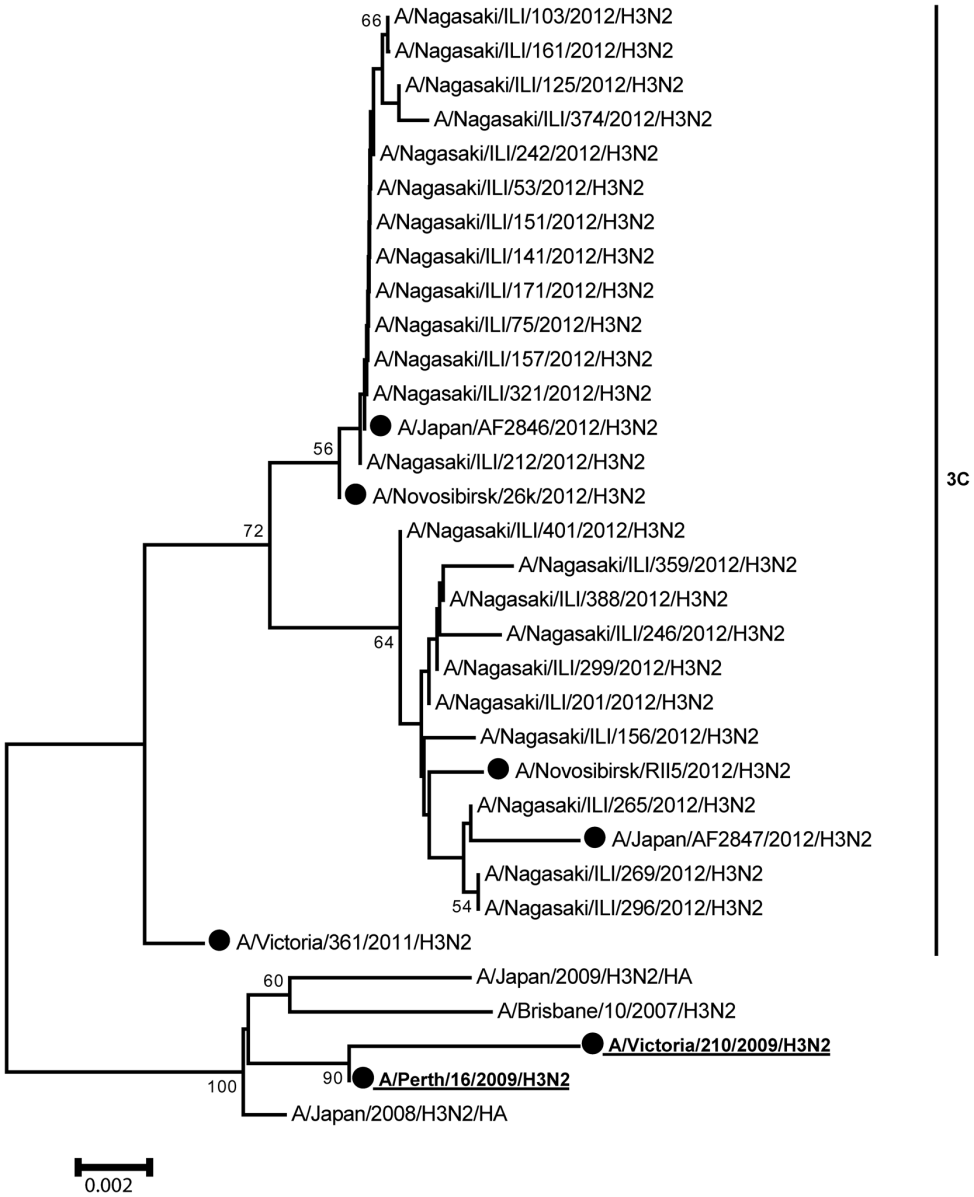
Phylogenetic tree of influenza A (H3N2) viruses in Nagasaki, Japan (2011–2012 season). Numbers at nodes indicate confidence levels of bootstrap analysis with 1,000 replicates as percentage values. Vaccine strains are boldfaced and in red, and reference strains are boldfaced and in black.

In total, 23 H3N2 viruses were characterised by phylogenetic analysis of the HA1 sequence. All sequences were clustered within the A/Victoria/361/2011 clade, which was genetically separated from the A/Victoria/210/2009 clade used in the 2011–2012 influenza vaccine (Figure 2).

## Discussion

In Japan, influenza A(H3N2) was the dominant circulating virus during the 2011–2012 influenza season. The estimates of the VE of TIV against medically attended, laboratory-confirmed influenza and medically attended, laboratory-confirmed influenza A were 5.3 and 16%, respectively. Despite their wide confidence intervals, our estimates of low VE were consistent with reports from other countries [4], [8], [9].

### Comparison with other Studies

In the 2011–2012 season, A(H3N2) was also the dominant strain in Europe, and the estimate of the VE of TIV against laboratory-confirmed influenza A was 23–29% [4], [8], [9]. These values were lower than those determined for the 2010–2011 season (VE = 56–58%), when A(H1N1)pdm09 was the dominant strain [18], [19]. Two reasons are proposed to explain this low influenza VE in Europe. First, a poor match between the TIV reference strain and the circulating A(H3N2) strain was reported [20], [21]. A substantial proportion of circulating viruses showed reduced reactivity against A/Perth/16/2009, the vaccine virus used for the 2011–2012 northern hemisphere seasons [21]. Second, a decrease in the influenza VE in the late phase of the season was observed. In the UK, VE against influenza A(H3N2) decreased from 43% in the early phase (Oct 2011–Jan 2012) to 17% in the late phase (Feb 2012–Apr 2012) [4]. Additionally, the VE against influenza decreased from 37% to 19% in Spain [8]. The late peak of influenza in the season and waning immunity may have reduced the overall VE [4], [8], [9].

In Japan, the National Institute of Infectious Diseases reported that 71% of subtyped strains were A(H3N2), 0.2% were A(H1N1)pdm09, and 28% were B based on the national surveillance of this season [14]. HI assays using post-infection ferret antiserum raised against the vaccine virus recommended for the 2011–2012 influenza vaccine, A/Victoria/210/2009(H3N2), showed that 34% of the test viruses had a reduction in HI titre of eight-fold or more [14]. In our phylogenetic analysis, all sequences of A(H3N2) viruses were considerably separated from the vaccine reference strain. The vaccine strain mismatch partially explains the low influenza VE in Japan. Additionally, despite the limited sample size, a decreasing trend in VE was observed in our setting; specifically, the adjusted VE against influenza A was 21.7% in the early phase and 8.9% in the late phase. The low VE in Japan may be explained by the combination of the vaccine strain mismatch and the waning of protection, as is the case in Europe.

### Possible Effect of NIRVs on Influenza VE Estimates in Test-negative Case-control Studies

In the current study, influenza VE was estimated using all influenza-negative controls and compared with that estimated using RV-negative controls and NIPV-positive controls. Although the confidence intervals were wide and overlapping, the point estimate was highest when we used NIRV-positive controls, followed by all influenza-negative controls and RV-negative controls. This finding was consistent with the hypothesis recently proposed by Cowling et al [6]. According to their theoretical discussion, people naturally infected with influenza are protected from subsequent NIRV infection because influenza infection induces a short-term, non-specific immunity, whereas vaccinated people are protected from influenza infection but have a higher risk of NIRV infections. In fact, Cowling et al conducted a randomised controlled trial and demonstrated that TIV recipients had an increased risk of NIRV infections [7]. If the hypothesis is true, NIRV-positive cases are more likely to have been vaccinated for influenza; thus, in the test-negative case-control study, estimates of VE using the NIRV-positive controls are higher than those using all influenza-negative controls and those using RV-negative controls. A supporting finding was reported in Australia; specifically, Kelly et al conducted a test-negative case-control study on children and demonstrated that the influenza VE was 58% when all influenza-negative controls were used and 68% when NIRV-positive controls were used [22]. On the other hand, Sundaram et al recently reported that the VE did not differ when they used all influenza-negative controls and RV-negative controls [23].

Test-negative case-control studies estimate VE by comparing the vaccination status of influenza test positives with that of test negatives that include NIRV-infected cases [3], [5]. This study design allows for the collection of appropriate controls that are derived from the same source population as the cases. One of the most important assumptions in this design is that the controls are drawn from the population without consideration of their vaccination status [6], [24]. If the risk of NIRV infection is high among the people vaccinated for influenza, the inclusion of NIRV-positive cases in the test-negative control group overestimates the true VE; this phenomenon may be what we observed in the current study. However, our findings do not provide conclusive evidence regarding the effect of NIRVs on VE estimates. Also, it must be noted that NIRV positivity in nasopharyngeal samples does not necessarily indicate NIRV disease [25]. Further investigations are needed to evaluate the validity of including NIRV-positive cases in test-negative case-control studies.

### Usefulness of RIDT for Influenza VE Studies

In our previous study, we demonstrated that the test-negative case-control study using RIDT provides rapid estimates of influenza VE in clinical settings [10]. In the current study, we confirmed that the influenza VE estimate using RIDT results (adjusted VE = 5.2%) was almost identical to that using PCR results (adjusted VE = 5.3%). Although the use of RIDT tends to underestimate the true VEs [5] and does not consider NIRV infection status, the test-negative case-control study using RIDT is a reliable assessment tool for influenza VEs.

### Limitations of the Study

Our study has limitations because of the nature of observational study designs [10]. The vaccination history was taken from the questionnaire and electronic medical records only. Recall biases may have affected our VE estimates. Although all potential confounders were considered, unmeasured confounders, such as socioeconomic status, may have remained. The confidence intervals of our VE estimates were wide because of the small sample size. However, the VE point estimates were low and consistent with other studies. We therefore believe that the increase in the number of samples does not fundamentally change our conclusions. On the other hand, larger sample sizes and longer study periods are required in future studies to assess the age group specific- and season specific-effects of NIRVs on VE estimates.

### Conclusions

The influenza VE was limited in Japan during the 2011–2012 season. The vaccine strain mismatch and the waning of protection may explain the low VE. Our study suggested that the inclusion of NIRV-positive cases in the control group may affect VE estimates in test-negative case-control studies. Further investigations are warranted to identify an appropriate control group in this study design.
